# Adapting to climate change: Reflections of peasant farmers in Mashonaland West Province of Zimbabwe

**DOI:** 10.4102/jamba.v11i1.571

**Published:** 2019-03-19

**Authors:** Tinashe M. Mashizha

**Affiliations:** 1 Community Capacity Building Initiative Centre for Africa, Kadoma, Zimbabwe

## Abstract

Climate change is projected to have negative impacts on agricultural production in sub-Saharan Africa and this is likely to continue for decades, unless adaptation measures are implemented. The changing climate is a global challenge to sustainable livelihoods and economic development. Peasant farmers in Zimbabwe depend entirely on rain-fed agriculture, a situation that makes agriculture and rural livelihoods vulnerable to climate change. This paper discusses the findings of the study carried out in the Zvimba District amongst peasant farmers on their knowledge of climate change impacts and adaptation strategies. Semi-structured interviews, observations and document analysis were used as methodologies for data collection for the study. Purposeful sampling technique was applied to 40 peasant farmers. Qualitative data from interviews and focus group discussion were analysed using context analysis. Households acknowledged that rainfall amount has decreased over the last 30 years. Such changes have reduced agricultural productivity, and in response, communities have developed multiple adaptation strategies such as harnessing social capital, crop and livelihood diversification, engaging in small businesses and water harvesting for livestock keeping. The study concludes that there is a need to recognise the validity of indigenous knowledge and an inventory should be created for future use. The paper notes that diversification of adaptive strategies is vital for sustainable livelihood in a changing climate.

**Keywords:**

climate change; adaptation; Zimbabwe; livelihood diversification.

## Introduction

Climate change is real and its occurrence in Zimbabwe is no longer debatable. Climate change is evident, the effects and impact are being felt and threaten sustainable human development (Kurjia, Nanja & Stern 2011). Studies have shown that climate change is a global externality that negatively impacts households and communities and that its potential to disorganise economies is real; thus, its challenge at globe, national and local levels can no longer be denied (Mashizha et al. 2017b; Sultan & Gaetani 2016). Zimbabwe is already experiencing the effects of climate change, notably rainfall variability and extreme events and these conditions combined with warming trends are expected to negatively impact the economy and the livelihoods of the poor as they depend mostly on rain-fed agriculture (IIED 2012). The International Panel on Climate Change (IPCC) reported that Africa will experience increased water stress, decreased yields (because of the reliance on rain-fed agriculture), increased food insecurity and malnutrition as a result of the changing climate. As such, adaptation to climate change is crucial for sustainable food security and economic development. To reduce the economic and social impact of climate change in Zimbabwe, focus has to be on adaptation to the changes that are necessary to thrive in changing climatic conditions (Chikonzi, Murwendo & Simba 2013). Adaptation strategies assist smallholder farmers to achieve their food, income and livelihood security and it allows them to maintain livelihoods in the face of climate change. Therefore, farmers can reduce the potential damage by making tactical responses to climate change (Tabbo & Amadou 2017). It is in this context that this study was conducted to establish impacts of climate change and how subsistence farming communities are coping or adapting to associated climate change risks. It is vital to build adaptation capacities amongst subsistence communities to minimise the effects of climate change. This paper sought to fill this knowledge gap by exploring the impact of climate change and variability on subsistence farming communities and the adaptation strategies devised by peasant farmers in Zvimba, Mashonaland West Province of Zimbabwe.

### Concept of climate change adaptation

The concept of adaptation is not new as it is widely used. Adaptation remains to be a common practice that has been widely used before (Burton, Diringer & Smith 2006). Burton et al. (2006) went on to further explain that human beings are amongst the most adaptable animal species on earth. Humans have been adjusting to changes in the environment few decades ago. Adaptation in the context of climate change is viewed as a means of strengthening resilience of individuals and systems to climate change and climate vulnerability (Elmum, Modise & Marr 2017). Apparently, adaptation has most often been assessed in the context of vulnerability to climate change. As a result, adaptation to climate change is usually preceded by analysis of perception of climate change as this is what spurs an individual or group to want to respond to perceived climate change or not (Elmum et al. 2017). Adaptation measures in agricultural practices include crop and livestock variation, community-based adaptation, water storage, irrigation, rainwater harvesting, water-conserving techniques and the use of drought-resistant crop varieties (Mugambiwa 2018).Current induced climate change experiences in developing countries are huge and causing devastating experiences to the poor (Dujardin, Hermesse & Dendoncker 2018). The huge challenge of this exposure to changing climate is exacerbated by high levels of sensitivity of social and ecological systems coupled with limited capacity of developing states to respond to the effects. With climate change, adaptation has gained new prominence and probably a new meaning. Climate change present not only threats to human lives and livelihoods but also risks to the economy and infrastructures (Mashizha, Monga & Dzvimbo 2017a). Developing countries need to invest in environmental practices that are resilient to climate change as it goes a long way in addressing the challenges and risks posed by climate change.

### Africa, the most vulnerable continent

Africa is epitomised as the most vulnerable continent to climate changes (Bwalya 2013; Masipa 2017). The continent will experience increased water stress, decrease in yields and increased food insecurity and malnutrition because of climate change (IPCC 2012). The African continent is most vulnerable to climate change and variability, increasing the hardships experienced by vulnerable communities (O’Brien et al. 2008; Scholtz 2011). By 2020, climate change will have impacted the lives and livelihoods of approximately 250 million people in Africa (Scholtz 2011). Extreme weather conditions such as droughts, floods and tropical storms are expected to increase in frequency and intensity across the continent (IPCC 2012). The continent will be affected because of its global position, its vulnerable populations and its poor land-use practices. A study in South Africa shows a 23% reduction in gross domestic product (GDP) as a result of climate change with an estimated 1.6% annual loss (Mthembu & Zwane 2017). South Africa will experience a 0.13 °C rise in day temperatures with dry land and smallholder farmers being mostly affected and reduction of rainfall will be extensive, ranging from 5% to 10%. Mashizha et al. (2017b) highlight that food production assessment indicates that domestic food production has already declined by 10% in several of the sub-Saharan countries. Climate change scenarios across multiple general circulation models show increases in Zimbabwe’s average mean temperature. Predictions show that the mean daily temperature will rise by 3 °C – 5 °C throughout the country and the mean annual temperature will rise by 2 °C – 4 °C (Mashizha et al. 2017a). However, it is important to understand that many of Africa’s problems result from factors other than climate change (Brazier 2015). Population growth, urbanisation, agricultural growth and land-use change present the effects; hence, developing countries need to address resource management issues urgently. Africa will feel the impact more because of slow technological changes within the continent and its economies depend more on agriculture (Mathews 2016; Mathews, Kruger & Wentink 2018). Climate change impact studies, although they are still uncertain on the frequency and severity of adverse weather events, have shown that the effects are significant for low-input farming systems, such as subsistence farming in marginal areas and because of socio-economic, demographic and policy trends have the least capacity to adapt to changing climatic conditions (Mutekwa 2009). Climate change is a serious threat to development and poverty reduction efforts in Africa (Mugambiwa & Dzomonda 2018; Mugambiwa & Tirivangasi 2017). The continent is facing various health risks associated with climate change and threaten to reverse development efforts and subject the continent to long-term poverty. With a temperature increase of 3 °C alongside the global warming anomaly, about 250–550 million people may be at risk of hunger with more than half of these people concentrated in Africa (Kangalawe & Lyimo 2013).Vulnerability of the African continent to climate change is a function of many factors including long-term poverty, illiteracy, political and ethnic conflicts, poor governance, lack of skills, weak technical institutions, poor and probably limited infrastructure and poor technological development (Ndaki 2014). Thus, Zimbabwe faces the risks associated with climate change and the country is at a very high level in terms of vulnerability.

### Adaptation and adaptive capacity in Zimbabwe

Adaptation is the process through which societies increase their ability to cope with an uncertain future, which involves taking appropriate action and making the adjustments and changes to reduce the negative impacts of the changing climate (Brazier 2015). The International Panel on Climate Change fifth assessment report indicates that countries have the potential of reducing the impacts of climate change through effective adaptation measures (IPCC 2012). Adaptation should be a concern to African countries as climate change impacts already have and will continue to negatively impact on needs of human beings that includes food, water and shelter. The World Bank (2011) asserts that vulnerable populations and societies such as the indigenous people, elders and children in the developing world will suffer more because of climate change. Africa’s adaptive capacity to climate change is undermined by several factors that range from limited understanding of the nature and consequences of climate change, farm members’ health status (particularly in relation to human immunodeficiency virus [HIV] and/or acquired immune deficiency syndrome [AIDS]), unemployment that is supposed to both complement and supplement agricultural incomes and poor rural infrastructure (Mutekwa 2009). Limited awareness about the nature and magnitude of climate change starts with researchers and academics. There are several climate change issues that have not yet been established with certainty that are important for agriculture, such as the time of onset of summer rainfall and the prevalence of dry spells within the rain seasons in southern Africa (Mutekwa 2009). It is against this background that adaptation involves changes to behaviour that includes planting of drought-resistant crops and changes to infrastructure. All stakeholders both at national and global level can contribute to climate change adaptation. Studies on adaptation point that each community has distinct levels of vulnerability and resilience and that each situation is different. Therefore, a blanket, top-down approach is not going to be successful (IIED 2012). The extended and complexity of climate change issues in Zimbabwe has seen organisations working together to better coordinate knowledge and helping communities to adapt to the impacts of climate change. In 2014, the Global Environmental Facility handed a grant of $3.9 million to scale-up climate change adaptation (UNDP 2016). In 2015, the Ministry of Environment, Water and Climate launched a project (scaling up adaptation in Zimbabwe), with a focus on rural livelihoods. The mentioned project seeks to scale-up climate change adaptation measures of rural communities and it targets to reduce the vulnerability of women (UNDP 2016). More to the above, Zimbabwe created the National Adaptation Plan (NAP) to climate change, which seeks to improve the resilience of cities to climate change impacts and ensures climate smart urban investments (UNDP 2017). National Adaptation Plan is a flexible process that builds on the country’s existing adaptation activities and helps integrate climate change into national decision-making (UNDP 2017). Zimbabwe’s vulnerability is related to lack of adaptive capacity and coping with additional stress posed by climate change. Adaptive capacity is typically limited by poverty, poor public and environmental health, weak institutions, lack of infrastructure and services, marginalisation from decision-making processes and planning procedures, gender inequality, lack of education and information, natural disasters, environmental degradation, reliance on rain-fed agriculture and climate-sensitive resources, and insecure tenure (IIED 2012). Thus, there is need for external support from national government agencies, to poor people who possesses the lowest levels of adaptive capacity.

### World efforts and challenges to adaptation

Adaptation to the current and anticipated impacts of climate change is not an easy task. Financing climate change adaptation remains a major challenge for developing countries, which are faced with many other development priorities that require financial investment. On the other hand, the international community has not been able to ensure predictable and sustainable adaptation funding sources for developing countries particularly the least developed countries (LDCs) (Ndaki 2014). The World Bank estimated that adaptation cost ranges from $70 to$100 billion per year (World Bank 2011). AFDB (2011), through its study on the cost of adaptation to climate change in Africa, concludes that adaptation costs in Africa are to the tune of $20–$30 billion per annum. Ndaki (2014) states that whilst these estimates are important in making advancing a strong case for availability of adequate adaptation funds, planning and budgeting, they have not been consensually agreed and that is where the challenge emanates. Financing adaptation programmes is a major constraint for developing countries, yet they are the most vulnerable group. More to the above, climate by itself is a complex phenomenon. Scholars such as Dessai (2009) argue that no one really knows how the climate is going to behave in 30 years. Hence, collective efforts are critical in addressing climate change. To date, the international community has made efforts in addressing this predicament. International organisations such as the IPCC are working hard to minimise the effects of climate change. Conventions such as the environmental summit in Rio de Janeiro and the Kyoto Protocol in Japan all sought to address and minimise the effects of the changing climate.

## Study area and data collection methods

The researcher used a qualitative research method for this study. Household survey was carried out using semi-structured and face-to-face interviews with participants conducted between April and July 2017. The questionnaire consists of methods of adaptation to climate change that farmers frequently used. The researcher allowed participants to compare the climate conditions of past 30 years with respect to the mean precipitation and temperature. If they had noted changes, they were asked actions in which they had taken to the perceived climate changes. The study used purposeful sampling technique, which is also known as purposive and selective sampling. This sampling technique enables the researcher to recruit participants who can provide in-depth and detailed information to ensure that research questions were answered. Palys (2008) argues that purposive sampling reaches a target sample quickly, economical and it gives better results. However, like any other sampling technique, purposive sampling has its own disadvantages. There is no equal chance for all the items of the universe being included in the sample (Palys 2008). The study was made up of three purposively selected villages, namely Kawondera, Gwamba and Tigere. The researcher focused on participants who were either aged 45 years and above. Age restriction was done to engage people who were capable of giving a comparative explanation of climate change between the periods when they were young. A total number of 40 households were interviewed and it comprised 23 women and 17 men. Two focus group discussions were conducted, which consisted of 11 people from different age groups. Focus group discussions were aimed specifically to gather and capture the information on the diversity of livelihood activities that reflect adaptive capacity. They were used to have an insight of people’s perceptions on climate change and how it affects food security and nutritional status of communities. Participant individual interviews were drawn from two staff at Agritex Department, one at Ministry of Environment, Water and Climate, three head men and one non-profit organisation (NGO) staff. Coding was used to structure the data. A phrase was assigned to capture whatever is salient. Qualitative data from interviews and focus group discussion were analysed using context analysis.

The study was carried out in Zvimba, a district located in Mashonaland West Province of Zimbabwe. The subsistence economy of Zvimba District is based on conservation farming and favourable crops grown in the area including maize, finger millet, ground nuts, vegetables and sorghum (Zimvac 2017). According to Zimstat (2012), Zvimba District has an estimated population of 67 591 based on the 2012 national census. The district is boarded by six other districts and its main town is Murombezi. The coordinates of the district are: 17° 42’ 0.00”S, 30° 12’ 0.00”E.

### Crop diversification as adaptation strategy

Households in Zvimba District have resorted to crop diversification as a way of minimising the risk of crop failure. Farmers are not only resorting to crop diversification but also resort to diversification of livelihood activities in a bid to minimising impacts of food insecurity and vulnerability. From the focus group discussions conducted and household interviews, it emerged that small-scale farmers in Zvimba District are responding to climate change by diversifying their range of crops and adapting to short-season varieties and small grains. The researcher noted that drought-resistant crops that include sorghum, rapoko and finger millet are most favoured by many peasant farmers. This study noted that growing of small grains is one of the local adaptation strategy to the impact of climate change as rural farming is now affected by unreliable rains. The participants considered cultivation of drought-resistant crops as an important tool for addressing crop failures. This trend clearly portrays that households understand the importance of cultivating small grains and drought-tolerant crops. Crops are grown in diverse mixtures, aiming at increasing farm productivity and avoiding the risk of crop failures. The argument of this research is that growing of small grains and drought-resistant varieties ensures that under stressful environments, households have sufficient grains to survive. Researches in other parts of Africa demonstrate that farmers’ adaptations to climate change variability is centred on diversification to take advantage of the differential effects that a given climate event or condition might have on different crops and activities during the year (Fisher et al. 2015). For example, in rain-fed systems that are prone to drought, diversification of farm plot locations can take advantage of spatial variability in rainfall.

**FIGURE 1:**
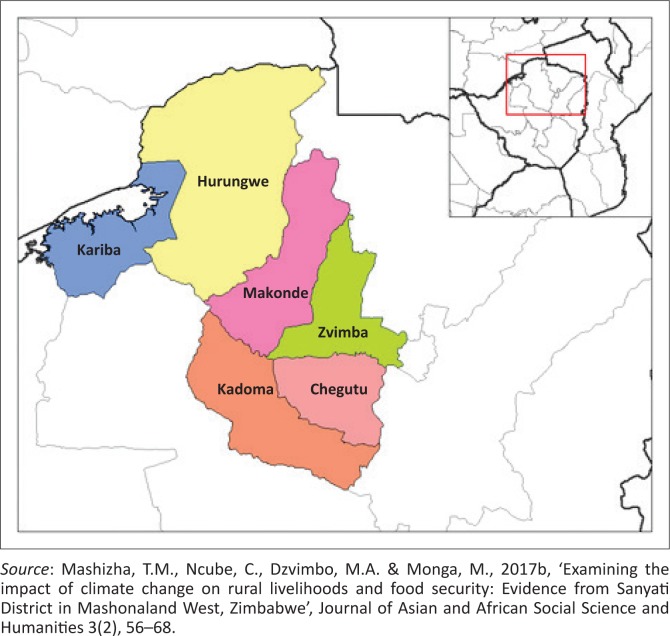
Map showing Zvimba District.

The researcher observed that every peasant farmer including those who claimed to have no knowledge on climate change have adapted to at least one strategy to cope with the changing climate. Respondents agree that climate change is eroding some of the coping strategies they used to implement decades ago and that they need to re-examine the current land-use management. Although the most popular strategy of dealing with climate change is growing of short-season varieties and growing of small grains as indicated earlier before, the main issue that needs to be emphasised is to vary planting dates. Varying of planting dates ensures that all plants do not coincide with harsh climate conditions at the same time.

### Livelihood diversification as an adaptation strategy

Peasant farmers in Zvimba District have not only resorted to crop diversification to deal with the effects of climate change but also resorted into livelihood diversification. Livelihood diversification is now an important aspect that minimises the effects of climate change on food security and becoming an important adaptation strategy. Participants for this study indicated that more people were migrating to nearest towns such as Chegutu, Chinhoyi and Norton and these migrations were climate change–related ones. Murombedzi and its surrounding areas no longer receive adequate rainfall that sustains crop cycle; hence, farmers harvest insufficient grain. Thus, people are migrating to nearest town. Young and energetic people are going as far as South Africa and Zambia and their families depend on remittances to supplement agricultural incomes and buying of food commodities during drought periods. Other community members have resorted to artisanal mining in areas such as Gadzema in Chegutu and Chinhoyi, although they know very well that the activity is illegal and causes environmental degradation. From the total of 40 respondents selected, all respondents indicated that casual labour is a livelihood adaptation strategy against climate, 38 indicated selling of fruits (guavas), 37 indicated gathering of fruits (mazhanje), 36 indicated artisanal mining, 31 indicated migration, 24 indicated small businesses such as kiosks and 22 indicated petty business (selling of juice cards) as shown in Table 1. In times of severe droughts, households rely on food aid from NGOs and the government. This is supported by Kangalawe and Lyimo (2013), who in their research findings in Tanzania argue that diversification and adaptive strategies such as water harvesting for small-scale irrigation, integration of livestock and crop production, and non-farm activities are crucial to ensure sustainable livelihoods in a changing climate.

**TABLE 1:** Adapting strategies for Zvimba District.

Type of strategy	No. of respondents	
	Female	Male
Gathering of wild fruits (mazhanje)	21	16
Selling of fruits (guava)	23	15
Migration	14	17
Artisanal mining	20	16
Casual labour	23	17
Small business	15	9
Petty business (selling of juice cards)	8	14

No., number.

### Views and knowledge about climate change

In Zvimba District, people’s perceptions on climate change are based on the assessment of two components, that is, temperature and precipitation events experienced within the locality. It was important for the researcher to understand farmer’s perception on their local climate as it determines their adaption decisions. Changes in the local climate help’s households to make decisions at the right time to either change their practices to accommodate themselves to the changes or to otherwise to adapt. From the number of the selected respondents, 38 of those who noticed changes in climate indicated that rainfall patterns are now unpredictable and 35 indicated that there is an increase in dry spell. Interestingly, all respondents highlight that normal rainfall is becoming more frequent. The majority of respondents indicated that rain season used to begin in late October, and by Christmas time, households would be consuming green maize. However, the ongoing trend clearly shows that rain season is now beginning in early December as indicated in Table 2. With respect to temperature, peasant farmers observed that temperature is on an increasing trend, as evidenced by the high rate at which surface water sources dry up and the wilting of crops after the occurrence of a precipitation event occurs. Whilst climate change knowledge could help the farmers to be more innovative and receptive of the advice that they get from government and private institutions, the thrust is to assist the farmers to adapt to drought conditions that have always been affecting the farmers (Mutekwa 2009). The study also observed that traditional knowledge has been useful in adaptation strategies adopted by peasant farmers.

**TABLE 2:** Seasonal calendar for Zvimba District.

Activity	Original season	New season
Land preparation	June to September	July to October
Planting	October to December	December to January
Weeding	November to December	January to March
Harvesting	March to May	April to June
Processing	May to July	June to August

### Gender and climate change adaptation

In Zvimba, women and girls tend to be impacted more by climate variability. The notion that climate change presents a significant threat to human security especially women is no longer debatable and within the studied area, 40 of the respondents agreed to that. Participants indicated that in time of a drought, women stay at home with their families whilst men move away to look for other alternatives. It is within this period that women, presented with few options, find ways to earn a living. Participants from the Ministry of Environment, Water and Climate Change indicated that climate change is and will continue to exacerbate the gender dimensions of vulnerability. What makes women to be more vulnerable is the fact that they constitute the majority of peasant farmers who depend heavily on rain-fed agriculture, which is sensitive to the changing climate. Madzwamuse (2010) supports this as he argues that 70% of women in Zimbabwe are smallholder farmers who depend on rain-fed agriculture and climate-sensitive resources. This study noted that women are not periodically involved in household’s decisions. Therefore, women are left without cash savings or any other assets to sell in order to restock or buy basic food items during a crisis. Like it is in many African countries, climate change is impacting negatively the livelihoods of women and it is reducing their economic base and opportunities. Interesting to note is that women are vulnerable to climate change (because they perform house chores, i.e. collecting of water and feeding the family) yet they are not fully represented and given a space in decision-making process on issues related to the changing climate. The planning and management measures should ensure that women are given space to be champions in the planning of climate change and disaster risk management. Women must be captains in managing environmental protection programmes and should be given maximum support. Future planning must incorporate a gender-sensitive perspective, which requires an understanding of the ways in which climate change can intensify pre-existing inequalities between men and women (Chagutah 2010).

### Climate change and food security

Respondents from the study area reported that agricultural productivity has declined. Of the respondents, 33 respondents indicated that agricultural yields have declined and this is particularly caused by unpredictable weather patterns. However, seven respondents shared a different view as they blame the government for not planning properly for farming seasons and for corrupt activities. Farming inputs are distributed late and farmers cannot plan properly because of that. In fact, this jeopardises the timing and planting date of most peasant farmers. The agricultural sector is highly vulnerable to the effects of climate change yet it contributes immensely to the economy. Prevalence of crop pests and diseases was also reported to have increased and this poses further challenges to agriculture. The study findings show that a diverse set of crops have been abandoned and farmers now grow small quantities of other crops. Participants’ interviews with agric-extension workers in the area of study indicate that the decline in the growing of maize as a cash crop is associated with drought conditions. Although NGOs introduced mulching to reduce water loss and increase yields of maize, 11 of the selected respondents abounded the crop citing that the yields of the crop have declined over the years because of a decline in soil fertility and droughts. Scientific evidence supports peasant farmer’s views as it argues that warmer temperatures lead to phenology and shortening the growing season which contributes to a reduction in crop yield. The phenomena experienced in the study as noted and discussed suggest that food security and nutritional status of households is at threat. To strengthen food security and nutrition status of households in Zvimba, the government and other private stakeholders are supposed to initiate irrigation programmes which are sustainable and not short lived. Introducing of drip irrigation plays an important role in strengthening food security and at the same time saving water which focuses on the principles of sustainable development. This also strengthens household adaptive capacity when agriculture is negatively affected.

### Indigenous knowledge observation of climate change

Scientific evidence has shown changes in climate and experience of these changes are notable. Rivers are drying up, droughts are common and famers are experiencing shorter growing seasons with dry spells. Peasant farmers have noted the changes through observations of birds’ behaviour and the flowering pattern of particular trees which is used to dictate and predict droughts. Natural occurrences such as the appearance of certain birds, the abundance of fruits and flowering of certain plants to forecast weather is commonly used in Zvimba and it indicates the quantity of rain expected. The observations and information determine the timing of growing of crops and what to plant. Integrating indigenous adaptation methods with scientific ones can be effective if community members are allowed to participate fully. The adaptation strategies that were pointed out by community members in the study area are based on indigenous knowledge (IK) systems and shared knowledge. Farmers learnt lessons from previous climatic experiences and use them to enhance their adaptive capacity. Chanza (2015) stresses that one of the potential avenues for building resilient communities is through exploitation of knowledge, skills and experiences of people who experienced the impact of climate change. IK is embedded in local cultures through community interaction and contributes to the adaptive capacity when facing the impacts of climate change. Zvimba community has experts who are capable and have knowledge to undertake and maintain long-term climate observation. These experts are elders of the community who rely on traditional knowledge and oral history to observe and monitor weather changes. Many indigenous communities around the world have already reported some adverse impacts from changing climate conditions within their ecosystems on which they depend on (Saitabau 2014). Local people have wealth of knowledge which needs to be built on as this helps in making adaptation strategies to be locally appropriate. In the past decade, NGOs have introduced adaptation strategies that were short lived and not sustainable mainly because locals were left out. The successful implementation of adaptation strategies needs to come from the community whilst the private sector and NGOs act as supporting mechanism in coordinating them. In this regard, the bottom-up approach needs to be fully integrated at all cost. Peasant farmers in Zvimba are applying their local knowledge to innovate new adaptive measures in response to the changing climate. This highlights the importance of indigenous weather prediction knowledge as timely warning of impending events as one of the best strategies for mitigating impacts of such events.

**FIGURE 2:**
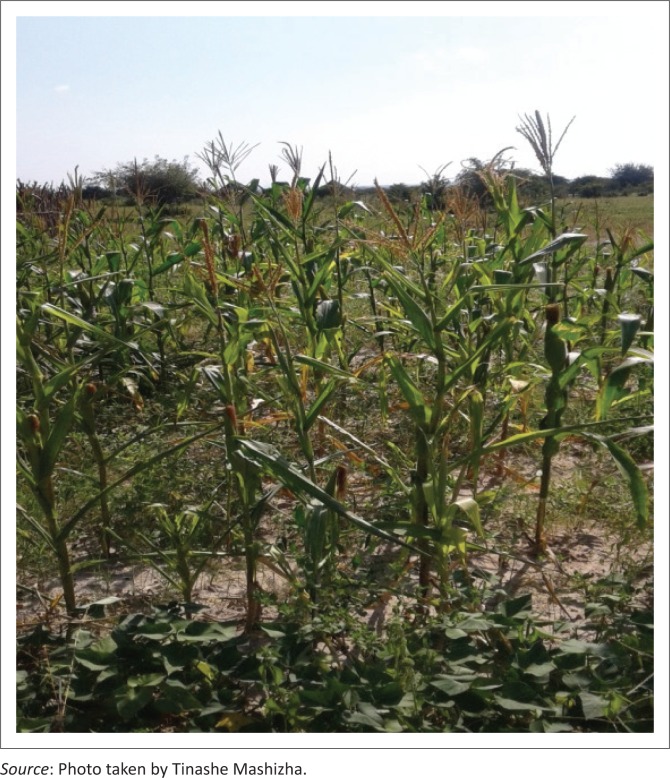
Rain-fed maize crops in Tigere village.

### Harnessing of social capital as adaptation strategy

This study revealed that there are traditional ways in which locals build mechanisms to cope and adapt to the changing climate. Zunde Ramambo, a traditional social mechanism designed to mitigate food security, is practiced in Zvimba as a way of coping with the effects of climate change. Zunde is a shona word that means a large gathering of people taking part in common activity or may refer to plenty of grain stored for future use by people in a particular community (Gukurume 2014; Mawere & Awuanh-Nyamekye 2015). Participant interviews from Agritex Department reviled that in Zvimba, there is land designated for the Zunde Ramambo where local people participate in the growing of food crops which will be distributed to those in need after harvest. The Zunde Ramambo system acts as a social safety net for vulnerable members of the community and it ensures that food security is guaranteed at all times. The practice ensures that agricultural duties that include planting, weeding and harvesting are done timely (Chanza & Ayal 2014; Mavhura 2017). The strategy adopted serves to strengthen the social capital nets of the community against potential risk of droughts. Zunde Ramambo is foresting community cohesion with different villages in Zvimba, and this allows communities to withstand the effects of droughts and famine. Peasant farmers are assisted to absorb shocks and be resilient because of community cohesion. Against this background, harnessing of social capital ensures that communities are protected in the event of drought and the Zunde Ramambo is a system which is significant in adapting to climate change.

## Conclusion and recommendations

The study has shown that agricultural activities of peasant farmers depend on rain-fed water. This suggests that agricultural activities are impacted negatively; livelihoods and food security in general are at threat. Zimbabwe is one of the most vulnerable countries and the exposure of climate change is widely exacerbated by poverty, unstable economy and limited mechanism put in place to cope effectively with the changing climate. If we are to closely look at preconditions that determine adaptive capacity, it is clear that Zimbabwe as a country is vulnerable because adaptive capacity is lacking. Adaptation strategies to climate change are supposed to be centred on the needs of peasant farmers. Peasant farmers are supposed to be encouraged to diversify to other economic activities as these strengthen household’s adaptive capacity. Other diversification activities complement each other and they ensure that food security at the household level remains good. Diversification is an important resilience tool and must be encouraged at all cost. The Zimbabwean climate change policy must recognise the applicability of IK and its value. Therefore, IK should be tapped and an inventory should be created for future use. IK indicators are useful and essential for crafting of frameworks related to climate change. The designing and implementation of adaptation activities are supposed to be based on social dimensions and perceptions of peasant farmers and not much on technical aspects. The integration of household’s perception ensures that there is an exchange of climate change information which is useful and important between donors and other stakeholders involved in the implementation of adaption projects. The study noted that there are peasant farmers who are still ignorant to climate change. Therefore, the study recommends the need of climate change campaigns informing communities about the impacts of climate change and possible adaption strategies they can carry out. Campaigns will also help to promote already adopted strategies.
